# Structural and functional brain asymmetries in the early phases of life: a scoping review

**DOI:** 10.1007/s00429-021-02256-1

**Published:** 2021-03-18

**Authors:** Patrizia Bisiacchi, Elisa Cainelli

**Affiliations:** 1 Department of General Psychology, University of Padova, Via Venezia, 8, 35121 Padova, Italy; 2 Padova Neuroscience Centre, PNC, Padova, Italy

**Keywords:** Lateralization, Hemispheric specialization, Neonate, Premature, Foetus, Newborn

## Abstract

Asymmetry characterizes the brain in both structure and function. Anatomical asymmetries explain only a fraction of functional variability in lateralization, with structural and functional asymmetries developing at different periods of life and in different ways. In this work, we perform a scoping review of the cerebral asymmetries in the first brain development phases. We included all English-written studies providing direct evidence of hemispheric asymmetries in full-term neonates, foetuses, and premature infants, both at term post-conception and before. The final analysis included 57 studies. The reviewed literature shows large variability in the used techniques and methodological procedures. Most structural studies investigated the temporal lobe, showing a temporal planum more pronounced on the left than on the right (although not all data agree), a morphological asymmetry already present from the 29th week of gestation. Other brain structures have been poorly investigated, and the results are even more discordant. Unlike data on structural asymmetries, functional data agree with each other, identifying a leftward dominance for speech stimuli and an overall dominance of the right hemisphere in all other functional conditions. This generalized dominance of the right hemisphere for all conditions (except linguistic stimuli) is in line with theories stating that the right hemisphere develops earlier and that its development is less subject to external influences because it sustains functions necessary to survive.

**Supplementary Information:**

The online version contains supplementary material available at 10.1007/s00429-021-02256-1.

## Introduction

Asymmetry characterizes the brain, with the right frontal and the left occipital lobes extending across the midline, a phenomenon often called the Yakovlevian torque (LeMay 1976). Asymmetries are present at all levels of structure and function, including regional volumes (Esteves et al. 2019), cortical thickness (Kong et al. 2018), connectivity (Thiebaut de Schotten et al. 2011), cellular and molecular organization (Chance 2014), neurite density (Schmitz et al. 2019) and surface area and gyrification (Chiarello et al. 2016). The most studied asymmetrical function is language, processed in more than 80% of individuals in the left hemisphere (Esteves et al. 2020). This hemispheric specialization appears to be functionally relevant, having been demonstrated to be advantageous in the execution of verbal tasks regardless of the direction of asymmetry (Hirnstein et al. 2014). Processing asymmetry has been observed in a broad range of functions, such as episodic memory (Habib et al. 2003), pseudoneglect (Zago et al. 2017), emotional valence (Brunoni et al. 2016), impulsivity (Gordon 2016), risk-taking (Telpaz and Yechiam 2014), and face processing (Zhen et al. 2015).

However, the process of specialization in one cerebral hemisphere (lateralization) is poorly understood. The hemispheric specialization is grounded in intra-hemispheric white matter connections, supported by associative bundles and inter-hemispheric connections between cortical areas located in mirrored positions (homotopic) through the corpus callosum fiber tracts (Ocklenburg et al. 2016). The possibility of associating these structural characteristics with functional correlates has only become possible relatively recently, with the advent of non-invasive neuroimaging methods. However, anatomical asymmetries explain only a fraction of functional variability in lateralization, and this may be associated with the fact that structural and functional asymmetries develop at different periods of life and in different ways.

The presence of asymmetries early in development have been investigated by observing foetuses’ or neonates’ movements, one of the most important manifestations of lateralization. Behavioral studies using ultrasound (US) observations of arm movements suggest the existence of motor lateralization as early as 12–27 weeks of gestation (McCartney and Hepper 1999). Furthermore, evidence for motor lateralization in the neonatal period has been associated with handedness (Cioni and Pellegrinetti 1982) and grasping strength (Tan et al. 1993).

Structural asymmetries were established in the foetal period in the 1970s, when neuropathological studies highlighted some larger areas (Heschl’s gyrus and planum temporale) in post-mortem foetuses on the left side (Witelson and Pallie 1973; Chi et al. 1977; Wada 1977).

In recent years, functional image-mapping techniques have emerged as a more sophisticated methodology, allowing researchers to study in vivo brain development; hemispheric asymmetries throughout the life span have been reported, with leftward and rightward asymmetries changing among brain structures at various ages (Matsuzawa et al. 2001; Andescavage et al. 2017). The emergence of asymmetries in the temporal lobes’ morphological development has been described as a major sign of lateralization. The most prominent asymmetry involves the peri-Sylvian region and superior temporal sulcus. Interhemispheric differences have been noted in newborn and young infants (Seidenwurm et al. 1985), with dynamic changes through childhood and adulthood (Sowell et al. 2002; Shaw et al. 2009; Fu et al. 2020).

However, most neuroimaging studies have been conducted in children older than age 4 due to the inherent challenge of acquiring data from younger infants. Furthermore, most studies have focused on cortical structures, while deep subcortical grey and white matter have been neglected. Thus, important questions about early brain maturation and hemispheric asymmetries remain unaddressed.

The third trimester of gestation and the neonatal period are the most important developmental periods for the formation of cerebral pathways in terms of path finding, target selection, and growing into the cortical plate (Suppiej et al. 2012). An essential feature of the third trimester of gestation is the transient organization of neuronal circuitry and foetal brain lamination (Kostović and Judaš 2006). This transient organization is supported by the subplate’s presence, the most prominent lamina on foetal brain histology, known to disappear at the end of the first year of postnatal life. At this stage of development, the major foetal zones are the cortical plate, subplate, intermediate zone, germinal matrix, deep grey nuclei, and ventricles.

The third trimester comprises the period between 20 and 45 weeks’ gestation; based on the major characteristic of the transient pattern of organization, it can be divided into four broadly defined phases: foetal (below 24 post-conception weeks [PCW]), early preterm (24–32 PCW), late preterm (33–35 PCW), and neonatal phases (36–45 PCW).

In this work, we aim to provide an overview of the evidence on cerebral asymmetries in the early development stage. We will review all published articles on the neonatal period (1–28 days of life, 36–45 PCW) and the third trimester of gestation (studies on foetuses during pregnancy and infants born preterm in the absence of medical or neurological complications). Premature infants are in vivo models of foetuses in the third trimester of gestation. However, in the absence of medical or neurological complications, “healthy” premature infants do not exist: Prematurity is a risk factor per sé. Therefore, studies on premature infants will be analyzed separately.

## Methods

Scoping reviews are ideal to determine the coverage of a body of literature on a given topic and give a clear indication of the volume of literature and studies available as well as an overview (broad or detailed) of its focus (Munn et al. 2018). Furthermore, scoping reviews can report on the types of evidence in a specific field and how further research may be done on a more specific question. According to the general purpose of scoping reviews, we aimed to identify and map the available evidence on hemispheric asymmetries in the earliest developmental stages.

Our scoping review focused on published works conducted on the first phases of brain development in the third trimester of gestation (studies on foetuses and infants born preterm) and the neonatal period (1–28 days of life or up to 45 PCW). Studies on at-risk populations or children suffering from pathological conditions were not included; prematurity is an at-risk condition. Still, studies on premature infants were included regardless of whether participants reported additional medical or neurological complications (e.g., intrauterine growth restriction, genetic syndromes, neonatal encephalopathy, intraventricular hemorrhage, periventricular cystic leukomalacia, or the occurrence of seizures).

The scoping review was performed using the PRISMA-ScR checklist for preferred reporting items for systematic reviews and meta-analysis extension for scoping reviews (Tricco et al. 2018). The resources obtained from this study are available as supplementary material.

## Assessment of methodological quality

In the current study, we included all papers investigating hemispheric asymmetries in foetuses and premature and full-term neonates, regardless of the technique, methodology of acquisition, pre-processing, or processing algorithms used. Where possible, structural and functional studies were divided into separate sections.

According to the scoping review aims, we did not produce a critically appraised and synthesized result/answer to a particular question; we rather aimed to provide an overview of the evidence in the field. Therefore, we did not perform a structured assessment of methodological limitations or risk of bias of the studies included. However, each work was assessed critically, and the weaknesses and strengths guided us in interpreting the results.

## Eligibility criteria

To be included in the review, papers needed to measure hemispheric asymmetries. Peer-reviewed journal papers were included if participants were foetuses or premature or full-term infants from birth to 45 PCW or 28 days of life; cerebral asymmetries were measured independently of the methodology used; and they were written in English. Behavioral studies on motor lateralization, single cases, animal studies, or studies on at-risk populations or children suffering from pathological conditions were excluded (for the same reason, post-mortem studies were not included). We also excluded works that did not fit into the revision’s conceptual framework and those that did not include hemispheric asymmetries in the title, keywords, or abstract. Finally, studies on a wider range of ages than that selected were only included if the results at various ages were clearly stated and differentiated. No limitations were put on the publication year range.

## Information sources

We conducted our search in September 2020, and the search strategies were drafted by two independent neuropsychologists (P.B. and E.C.). We searched PubMed and SCOPUS (Elsevier API) bibliographic databases (which include most of the EMBASE database, https://www.elsevier.com/solutions/embase-biomedical-research). The search was conducted using the following string: (“asymmetry” OR “laterality”) AND (“cerebral” OR “brain” OR “hemispheric”) AND (“newborn” OR “neonatal” OR “preterm” OR “fetal” OR “foetal”), which returned a total of 2238 results on Scopus and 1712 on PubMed. There were no internal duplicates within either database. The final search results were exported for storage and remotion of duplications into the Mendeley bibliographic software package. External duplicates between the databases were removed from the list. The electronic database search was supplemented by screening the reference lists of each retrieved paper and scanning relevant reviews, obtaining an additional 10 works. A total of 2,403 results were screened.

## Selection of sources of evidence

**Fig. 1:**
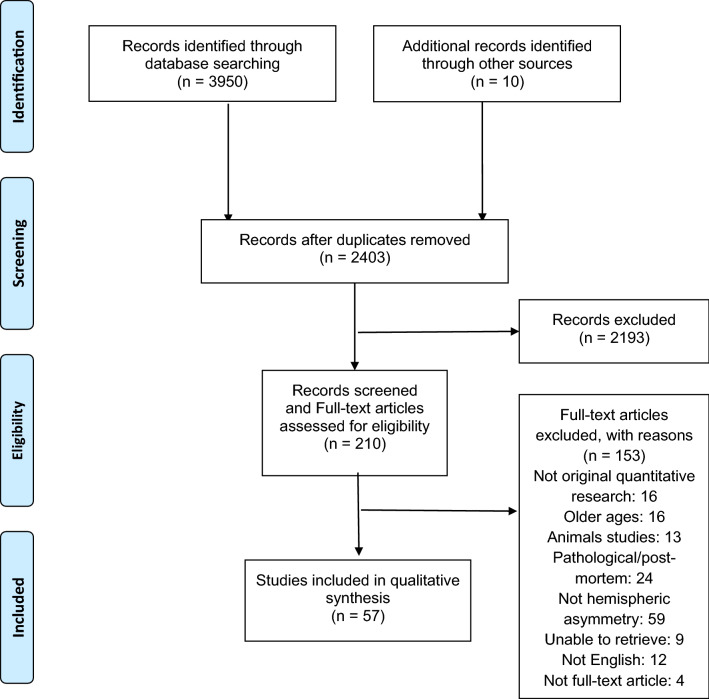
Workflow of the selection of sources of evidence

Figure 1 summarizes the following workflow.

The 2403 results were screened based on the article’s titles, and 2194 were excluded because they did not focus on cerebral asymmetries, were not original research, or were animal studies. The full texts of the remaining 210 papers were screened, and further exclusion criteria led to the exclusion of 153 additional works. Articles were excluded based on not being original quantitative research (*n* = 16), involving older participants (*n* = 16), being animal studies (*n* = 13), involving participants with pathological conditions or post-mortem studies (*n* = 24), not focusing on hemispheric asymmetries (*n* = 59), being irretrievable (*n* = 9), having only an abstract in English (*n* = 12), or having only the abstract available (*n* = 4). The final analysis included 57 studies.

## Data charting process and data items

A data-charting form was jointly developed by two reviewers (P.B. and E.C.) to determine which variables to extract. The two reviewers independently charted the data, discussed the results, and continuously updated the data charting form in an iterative process. Any disagreements were resolved through discussion between the reviewers. Data from eligible studies were charted using a standardized data abstraction tool designed for this study (see Appendix 1). The tool captured the relevant information on key study characteristics and all techniques used to investigate hemispheric asymmetries.

## Data items

The extraction form is comprised of general and specific characteristics of the articles: reviewer identity, date of reviewing, first author’s name, publication year, the title of the article, the journal, the technique used, paradigm and analysis (for functional studies), number of participants, age at recording, participants’ status (full-term, premature, or gestation), and a short description of results (see Appendix 1).

## Results

Of the 57 articles eligible for review, 33 were conducted in the neonatal period, 11 during gestation, and 10 before 40 post-conception age (PCA); 3 additional works cover both the neonatal period and the weeks before term PCA.

**Fig. 2:**
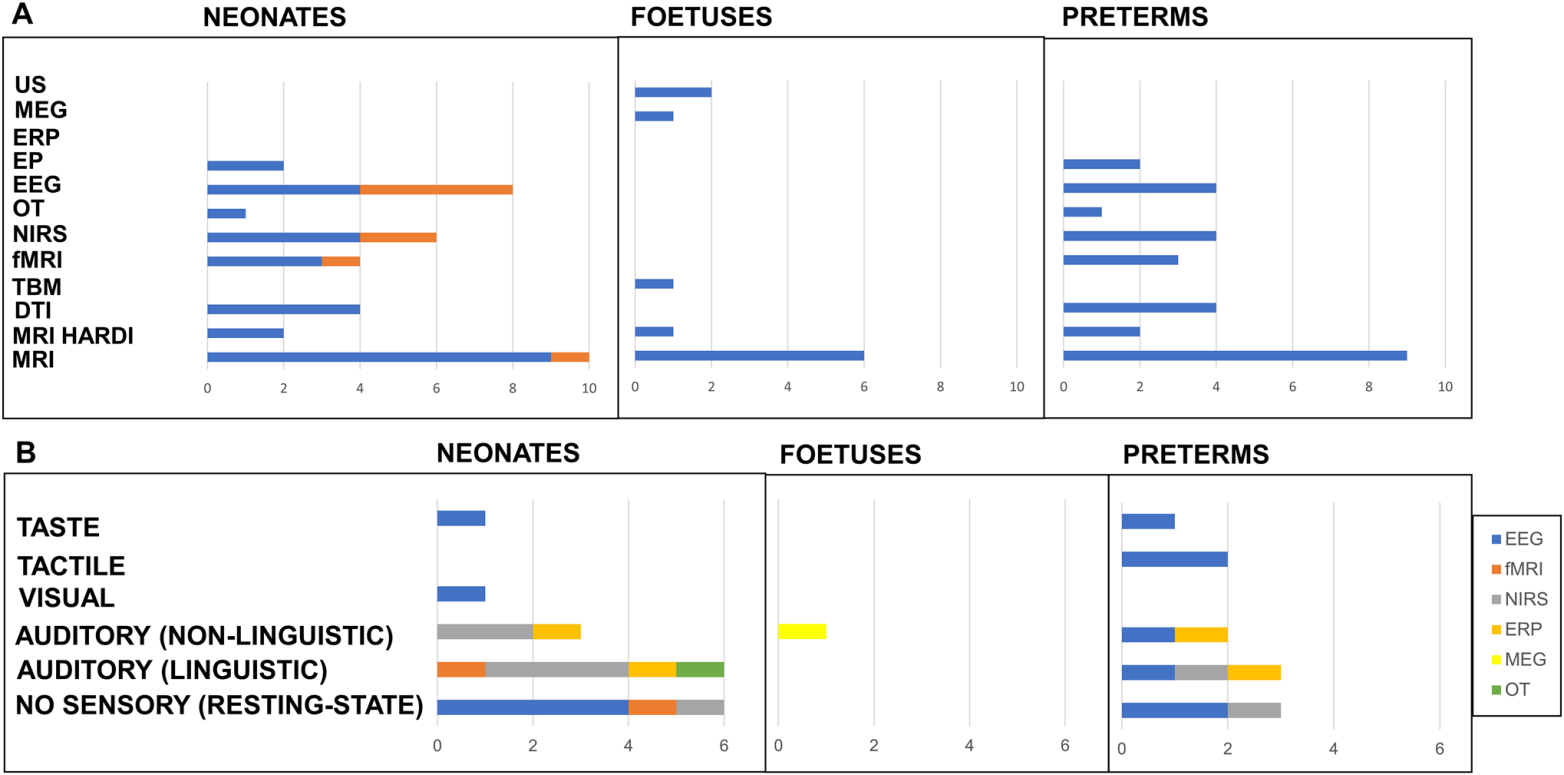
The number of studies and structural methodologies used in neonates (red represents studies on full-term neonates; blue represents studies on both full-term and preterm neonates at term PCA), foetuses, and preterms (Panel **a**). The number of studies and functional methodologies used in neonates, foetuses, and preterms (without sensory modality [resting state], auditory [linguistic and non-linguistic] visual, tactile, and taste stimuli) (Panel **b**). *DTI* diffusion tensor imaging, *EP* evoked potential, *EEG* electroencephalogram, *fMRI* functional magnetic resonance imaging, *HARDI* high-angular resolution diffusion imaging, *MEG* magnetoencephalography, *MRI* magnetic resonance imaging, *NIRS* near-infrared spectroscopy, *OT* optical topography, *PCA* post-conception age, *TBM* tensor-based morphometry, *US* ultrasound

Figure 2 shows an overview of the number of studies and methodologies used.

**Fig. 3:**
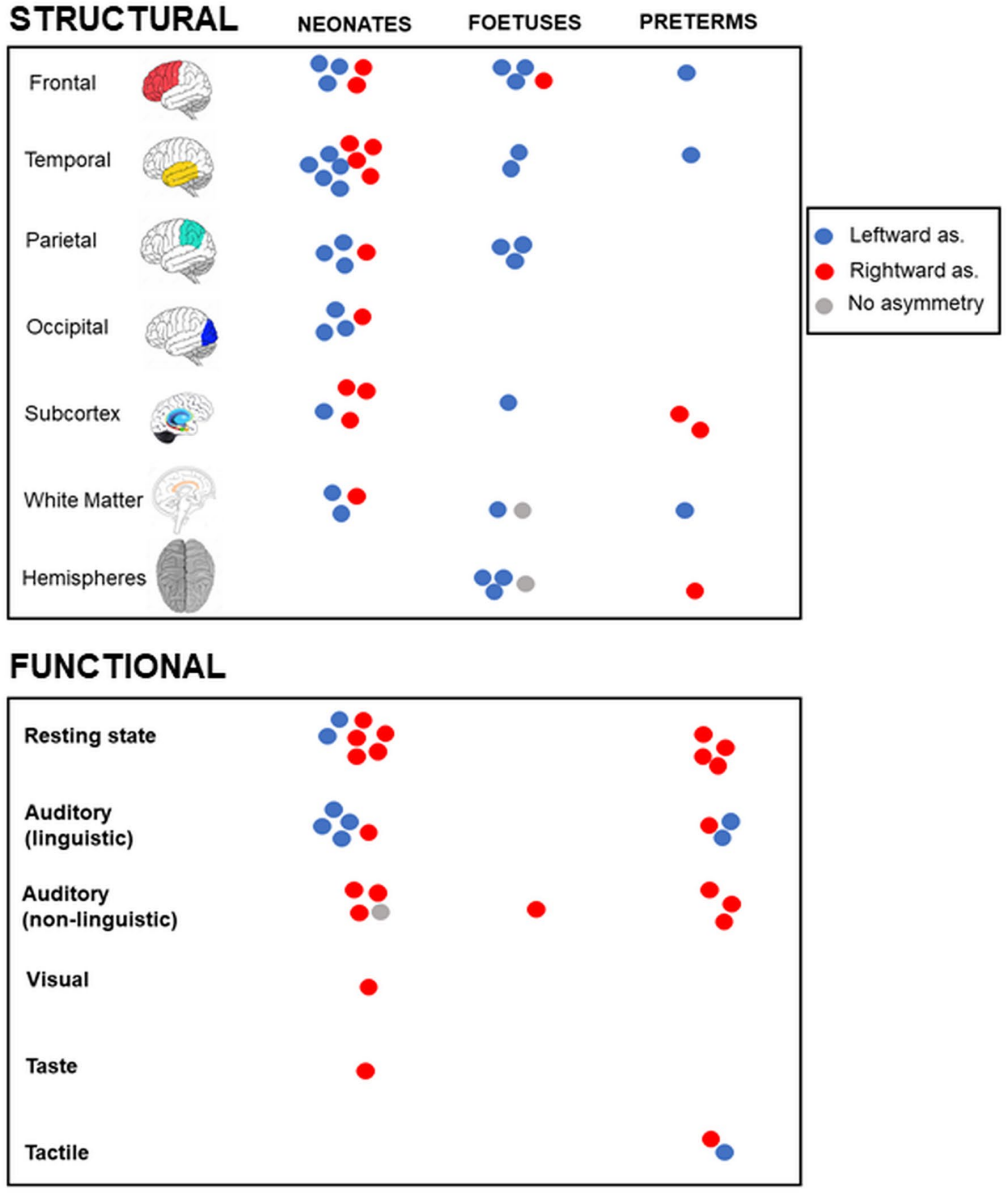
Overview of the findings on structural and functional brain asymmetries in neonates, foetuses, and preterms

Figure 3 shows an overview of the findings on brain asymmetries.

## Neonatal period

Thirty-six studies investigated hemispheric asymmetries in the neonatal period; 8 were mixed studies on preterm infants at term PCA and/or full-term infants, 3 of which covered both the neonatal period and the weeks before term PCA. This section contains the results obtained from full-term neonates; results obtained from premature infants at term PCA were reported separately in a dedicated paragraph. Articles are reported in Table 1.

**Table 1:** All studies investigating hemispheric asymmetries in the neonatal period in full-term neonates and healthy preterm infants as they reach term

Structural asymmetries in the neonatal period					
Authors, year	Technique	Participant number	Participant status	Age at recording	Study design
Gilmore et al. (2004)	3 T MRI DTI	20	Full-term	Newborn	Right/left and sex within-subjects comparison
Gilmore et al. (2007)	3 T MRI	74	Full-term	42.8 ± 1.6 PCA	Right/left within-subjects comparison
Thompson et al. (2009)	1.5 T MRI	32 full-term 184 preterm	Full-term (39 ± 1.2 GA) Preterm (27.6 ± 1.9 GA)	At term PCA	Right/left within-subjects comparison Preterm/full-term between-subjects comparison
Hill et al. (2010)	3 T MRI	12	Full-term	39 PCA	Right/left within-subjects comparison
Liu et al. (2010)	1.5 T MRI DTI	37.5 ± 1.5 PCA	27	30.0 ± 2.3 GA	Right/left and age within-subjects comparison
Ratnarajah et al. (2013)	1.5 T DTI	124	Full-term	5–17 days	Right/left within-subjects comparison
Li et al. (2014)	3 T MRI	73	Full-term	27.3 ± 13.1 days 1, 2 years	Right/left and age within-subjects comparison
Meng et al. (2014)	3 T MRI	73	Full-term	27.3 ± 13.1 days 1, 2 years	Right/left and age within-subjects comparison
Li et al. (2015)	3 T MRI	73	Full-term	27.3 ± 13.1 days 1, 2 years	Right/left and age within-subjects comparison
Wilkinson et al., (2017a, b)	3 T MRI HARDI of arcuate fasciculus	83	Full-term	40 PCA–28 years	Right/left within-subjects comparison Age between-subjects comparison
Wilkinson et al. 2017a, b	3 T MRI HARDI of thalamus	83 in vivo 11 post-mortem foetuses	Full-term foetuses	40 PCA–28 years Post-mortem foetuses	Right/left within-subjects comparison Age between-subjects comparison
Dean et al. (2018)	3 T MRI	143	Full-term	1 month	Right/left within-subjects comparison
Lehtola et al. (2018)	3 T MRI	68	Full-term	2–5 PCA	Right/left within-subjects comparison Age between-subjects comparison
Duan et al. (2019)	3 T MRI	595	Full-term	36.7–46.5 PCA	Right/left within-subjects comparison
Vannucci et al. (2019)	1.5 and 3 T MRI	121	Full-term	0–18 years	Right/left within-subjects comparison Age and sex between-subjects comparison
Functional asymmetries in the neonatal period					
Croweell et al. (1973)	EEG repetitive visual stimulation Spectral analysis	97	Full-term	–	Right/left within-subjects comparison
Molfese and Nunez (1976)	AEP	14	Full-term	Within 48 h of birth	Right/left within-subjects comparison
Fox and Davidson (1986)	EEG Taste stimuli Spectral analysis	16	Full-term	2–3 days	Right/left within-subjects comparison
Majnemer and Rosenblatt (1992)	SEP	9	Full-term	2–3 days 2 months 6 months	Right/left and age within-subjects comparison
Eiselt et al. (1997)	EEG Resting state Spectral analysis	12	6 full-term 7 preterm (< 32 GA)	3–8 days	Right/left within-subjects comparison Preterm/full-term between-subjects comparison
Scher et al. (1997)	EEG Resting state Spectral analysis	55 preterm 45 full-term	Preterm (≤ 32 GA) Full-term	At term PCA	Right/left within-subjects comparison Preterm/full-term between-subjects comparison
Pena et al. (2003)	Optical topography Infant-directed speech, reversed, and silence	14	Full-term	2–5 days	Right/left within-subjects comparison
Ehberrich et al. (2006)	1.5 T fMRI Cutaneous and proprioceptive stimulation	42 newborn 6 infants	Newborn infants (3–9 months)	Mean 42 (38–49) PCA	Right/left within-subjects comparison Newborn/infants between-subjects comparison
Telkemeyer et al. (2009)	EEG NIRS Structured non-speech stimuli	34	Full-term	2–6 days	Right/left within-subjects comparison
Kotilahti et al. (2010)	NIRS Infant-directed speech and music	13	Full-term	37.3–42.3 PCA	Right/left and condition within-subjects comparison
Gonzales et al. (2011)	EEG Resting state Connectivity	21	7: 33–34 7: 38– 39 7: 38–39 GA	39–40 PCA 39–40 PCA 44–45 PCA	Right/left within-subjects comparison Age between-subjects comparison
Minagawa-Kawai et al. (2011)	NIRS Auditory stimuli (various durations)	29	Full-term	0–5 days	Right/left and condition within-subjects comparison
Perani et al. (2011)	fMRI Female voice Connectivity DTI	15 full-term adults	Full-term Adult	2 days	Right/left within-subjects comparison Age between-subjects comparison
Lin et al. (2013)	FDNIRS DCS Resting state	70	55 preterm (24–42 GA) 15 full-term	3.6 weeks	Right/left within-subjects comparison Preterm/full-term and sex between-subjects comparison
Myers et al. (2012)	EEG Resting state Spectral power Burst detection	252	171 preterm 81 full-term	35–52 PCA	Right/left and preterm/full-term between-subjects comparison
Bouchon et al. (2015)	NIRS Artificial and random grammar	24	Full-term	1–3 days	Right/left within-subjects comparison
Kwon et al. (2015)	3 T fMRI Resting state Connectivity	26 preterm 25 full-term	Preterm (500–1500 g) Full-term	At term PCA	Right/left within-subjects comparison Preterm/full-term between-subjects comparison
Arimitsu et al. (2018)	NIRS Phonetic changes	80	60 preterm (26–41 GA) 20 term	33–41 PCA	Right/left and Age between-subjects comparison
Barttfeld et al. (2018)	1.5 T fMRI Resting state	24	Full-term	41.1 ± 1.08 PCA	Right/left within-subjects comparison
Corsi-Cabrera et al. (2019)	EEG Resting state Spectral analysis	60	Full-term	41, 42, 43, 44, 45 PCA	Right/left within-subjects comparison Age between-subjects comparison

*AEP* auditory-evoked potentials, *DCS* diffuse correlation spectroscopy, *EEG* electroencephalogram, *FDNIRS* frequency domain near-infrared spectroscopy, *GA* gestational age, *HARDI* high-angular resolution diffusion, *MRI* magnetic resonance imaging, *PCA* post-conception age, *SEP* somatosensory-evoked potentials

The number of studies and methodologies used are shown in Fig. 2 (Panel A and B, “neonates”).

The following paragraphs contain the results of studies on structural and functional asymmetries. Results obtained for premature infants will be discussed separately at the end.

## Structural asymmetries in the neonatal period

Sixteen studies reported structural asymmetries in the brains of full-term neonates. These asymmetries were variable across regions.

Most studies showed a rightward asymmetry of whole subcortical grey matter (Dean et al. 2018) or the hippocampus (Thompson et al. 2009; Ratnarajah et al. 2013) and the putamen (Ratnarajah et al. 2013), but leftward asymmetry has also been reported (Gilmore et al. 2007).

A leftward asymmetry was found for white matter (Gilmore et al. 2007; Dean et al. 2018), but a study focused on arcuate fasciculus found a rightward asymmetry (Wilkinson et al. 2017b); the same author did not find asymmetries on the thalamic-cortical tracts (Wilkinson et al. 2017a).

Data focusing on cortex showed high variability. The temporal lobe is certainly the most investigated, but still with contrasting results: rightward asymmetry was found in the whole temporal lobe (Lehtola et al. 2019), in the superior temporal sulcus (Hill et al. 2010; Li et al. 2014; Lehtola et al. 2019), and the medial temporal and insula (Li et al. 2015). The other authors found a global leftward asymmetry (Gilmore et al. 2007) or one specific to areas such as the planum temporale (Hill et al. 2010; Li et al. 2014), entorhinal cortex, fusiform gyrus, insula (Ratnarajah et al. 2013), and the superior temporal sulcus (Duan et al. 2019).

Vannucci and colleagues (2019) found global rightward asymmetry for the frontal lobe, while other researchers indicated a leftward one (Gilmore et al. 2007; Li et al. 2015). Ratnarajah and colleagues found a rightward asymmetry in the cingulate cortex and the gyrus rectus, but a leftward one in the precentral gyrus (Ratnarajah et al. 2013).

A global leftward asymmetry was found for the parietal (Gilmore et al. 2007; Lehtola et al. 2019) and occipital (Gilmore et al. 2007; Lehtola et al. 2019; Vannucci et al. 2019) cortices or specific to the precuneus in the parietal lobe (Ratnarajah et al. 2013). Li and colleagues (2014) found a rightward asymmetry of the parieto-occipital sulcus.

A leftward asymmetry of the ventricles was found in two studies by Gilmore and colleagues (2004, 2007).

Results on structural brain asymmetries are shown in Fig. 3 (above, “neonates”).

## Functional asymmetries in the neonatal period

Studies on functional asymmetries can be conducted under stimulation or without sensory stimulation, reflecting the brain’s endogenous activity (resting state).

We found six studies investigating endogenous cerebral activity without sensory stimulation and 10 with sensory stimulation at full-term.

### Functional studies of endogenous activity

fMRIs demonstrated a stronger correlation of the medial temporal gyrus with other cerebral areas in the left hemisphere compared to the right (Barttfeld et al. 2018). Spectral analysis of the EEGs revealed significantly higher absolute power in the left central region and the right occipital and temporal areas (Corsi-Cabrera et al. 2020), the right centro-occipital areas (Eiselt et al. 1997), or a general rightward dominance in power below 13 Hz between 35 and 45 PCW (Myers et al. 2012). The behavioral state seems to affect spectral content in the right and left hemispheres: During active sleep, spectral power is higher in right posterior regions. During quiet sleep, spectral power is higher in the frontal areas of both hemispheres than the posterior (Scher et al. 1997). A rightward dominance in the temporal and parietal metabolism has been found using NIRS and diffuse correlation spectroscopy (Lin et al. 2013).

### Functional studies under stimulation

Speech stimuli (grammar and infant-directed speech) evoked leftward responses using NIRS (Kotilahti et al. 2010; Bouchon et al. 2015), optical topography (Peña et al. 2003), and auditory-evoked potentials (Molfese et al. 1976). By contrast, using fMRI and female voices, Perani and colleagues found a rightward asymmetry (2011). In addition, non-linguistic aspects of speech stimuli, such as prosody, evoked a rightward dominance based on fNIRS (Arimitsu et al. 2018).

Non-speech auditory stimulation evoked a rightward asymmetry using auditory-evoked potentials and NIRS (Telkemeyer et al. 2009) as well as auditory-evoked potentials alone (Molfese et al. 1976). By contrast, using NIRS, Minagawa-Kawai and colleagues did not find asymmetries (2011).

Finally, spectral EEG in response to rhythmic visual stimuli (Crowell et al. 1973) and taste (Fox and Davidson 1986) showed rightward asymmetry.

Results on functional brain asymmetries are shown in Fig. 3 (below, “neonates”).

## Gestational period

We found 11 studies exploring hemispheric asymmetries in foetuses during pregnancy (Table 2). All but one were structural investigations of hemispheric asymmetries. Articles are reported in Table 2.

**Table 2:** Studies investigating hemispheric asymmetries in foetuses during pregnancy

Structural asymmetries in the gestational period				
Authors, year	Technique	Age at recording	Participant number	Study design
Hering-Hanit et al. (2001)	US	20–22 GA	102	Right/left within-subjects comparison
Kivilevitch et al. (2010)	US	19–28 GA	406	Right/left within-subjects comparison
Kasprian et al. (2011)	1.5 T MRI	18–37 GA	197	Right/left within-subjects comparison Age between-subjects comparison
Rajagopalan et al. (2011)	1.5 T MRI	20–28 GA	38	Right/left within-subjects comparison Age between-subjects comparison
Scott et al. (2011)	1.5 T MRI	20.57–31.14 GA	39	Right/left within-subjects comparison Age between-subjects comparison
Habas et al. (2012)	1.5 T MRI	20–28 GA	38	Right/left within-subjects comparison Age between-subjects comparison
Rajagopalan et al. (2012)	Tensor-based morphometry	20.57–27.86 GA	38 (40 scans)	Right/left within-subjects comparison Age between-subjects comparison
Song et al. (2015)	3 T MRI HARDI	15 GA–3 years	23 (11 post-mortems, 12 foetuses)	Right/left within-subjects comparison Age between-subjects comparison
Andescavage et al. (2017)	1.5 T MRI	18–39 GA	166	Right/left within-subjects comparison Age between-subjects comparison
Vasung et al. (2019)	3 T MRI	16.43–36.86 GA	42	Right/left within-subjects comparison
Functional asymmetries in the gestational period				
Schleussner et al. (2004)	MEG 500 Hz	3rd trimester	38 (53 scans)	Right/left within-subjects comparison

*GA* gestational age, *HARDI* high-angular resolution diffusion, *MEG* magnetoencephalography, *MRI* magnetic resonance imaging, *US* ultrasound sonography

The number of studies and methodologies used are shown in Fig. 2 (Panel A and B, “foetuses”).

## Structural asymmetries in the gestational period

Data showed that during primary gyrogenesis, the right hemisphere undergoes cortical folding earlier than the left (Rajagopalan et al. 2011, 2012; Kasprian et al. 2011; Habas et al. 2012), with the appearance of the right superior temporal sulcus by 23 (Kasprian et al. 2011) or 24 gestational weeks (Habas et al. 2012). However, some specific areas of the left parahippocampal cerebral mantle have been reported to be larger on the left than the right (Rajagopalan et al. 2011), and a certain asymmetry pattern has been established with a longer left temporal lobe (Kasprian et al. 2011).

However, there is also contrasting evidence. A small but significant number of larger left hemispheres have been noted, both in cortical grey matter and deep subcortical structures (Andescavage et al. 2017). The larger volume of the left hemisphere was also confirmed by ultrasound examinations (Hering-Hanit et al. 2001; Kivilevitch et al. 2010).

Other cerebral areas, such as some regions of the frontal and parietal lobes (the inferior frontal gyrus and frontal operculum) showed leftward dominance (Rajagopalan et al. 2011, 2012; Vasung et al. 2020), while the orbitofrontal cortex showed a rightward one (Vasung et al. 2020).

A leftward hemispheric asymmetry was found in the inferior longitudinal fasciculus of the white matter. In contrast, asymmetry of pathways associated with higher-order cognitive functions, such as the arcuate fasciculus, was not observed (Song et al. 2015).

No significant differences in brain hemispheric symmetries were found by Scott and colleagues (2011).

Results on structural brain asymmetries are shown in Fig. 3 (above, “foetuses”).

## Functional asymmetries in the gestational period

In a unique study, researchers investigated the functional asymmetries in foetuses by recording magnetoencephalography during auditory stimulation (Schleussner et al. 2004). Researchers found a delay in the latency of the cortical auditory responses in the left hemisphere, suggesting earlier maturation of right brain areas.

Results on functional brain asymmetries are shown in Fig. 3 (below, “foetuses”).

## Premature infants before term PCA

We found 13 studies on hemispheric asymmetries in premature infants before term PCA. These studies showed great variability in methodologies and techniques.

In contrast to studies conducted in the neonatal period or during pregnancy, most studies on premature infants before term PCA were based on functional asymmetries. Articles are reported in Table 3.

**Table 3:** Studies investigating hemispheric asymmetries in premature infants before term PCA

Structural asymmetries in premature infants before term PCA					
Authors, year	Technique	Age at recording	Participant number	Gestational age	Study design
Dubois et al. (2008)	1.5 T MRI	31.1 ± 2.4 PCA	35	30.0 ± 2.5 GA	Right/left within-subjects comparison Age and sex between-subjects comparison
Dubois et al. (2010)	1.5 T MRI	31.5 ± 2.4 PCA	25	26–36 GA	Right/left within-subjects comparison Age between-subjects comparison
Guo et al. (2015)	1.5 T MRI on the hippocampus	Birth at term PCA	197	24–32 GA	Right/left and age within-subjects comparison
*Functional asymmetries in premature infants before term PCA*					
Eiselt et al. (1997)	EEG Resting state Spectral analysis	12	6 full-term 7 preterm (< 32 GA)	3–8 days	Right/left within-subjects comparison Preterm/full-term between-subjects comparison
Mento et al. (2010)	ERP Auditory oddball	35 PCA	34	24–34 GA	Cluster analysis
Lin et al. (2013)	FDNIRS DCS Resting state	70	55 preterm (24–42 GA) 15 full-term	3.6 weeks	Right/left within-subjects comparison Preterm/full-term and sex between-subjects comparison
Barlow et al. (2014)	aEEG orocutaneous st. Non-stimulation Spectral analysis	32 PCA	22	28.56 GA	Right/left within-subjects comparison Conditions between-subjects comparison
Maitre et al. (2014)	ERP Speech Psychomotor assessment	> 32 PCA	57	24–40 GA	Right/left and outcome within-subjects comparison GA/PCA between-subjects comparison
Song et al. (2014)	EEG Pulsed orocutaneous stimulation Spectral edge frequency (SEF)	32.2 ± 1.09 PCA	22	28.6 ± 2.1GA 1,230 ± 338 g	Right/left within-subjects comparison Condition between-subjects comparison
Kaminska et al. (2017)	EEG-MRI Auditory stimuli (click) Evoked potentials	30–38 PCA	30	26–36 GA	Right/left within-subjects comparison Age between-subjects comparison
Arimitsu et al. (2018)	NIRS Phonetic changes of speech	33–41 PCA	80	60 preterm (26–41 GA) 20 full-term	Right/left within-subjects comparison Age between-subjects comparison
Cainelli et al. 2019	EEG Resting state Connectivity	35 PCA	16	8: 23–28 GA 8: 34–35 GA	Right/left within-subjects comparison GA between-subjects comparison
Daneshvarfard et al. (2019)	EEG Repetitive syllables Spectral analysis	29.57–34.14 PCA	16	29.57–34.14 GA	Right/left within-subjects comparison Age between-subjects comparison

*aEEG* amplitude-integrated electroencephalogram, *DCS* diffuse correlation spectroscopy, *ERP* event-related potentials, *FDNIRS* frequency domain near-infrared spectroscopy, *GA* gestational age, *MRI* magnetic resonance imaging, *PCA* post-conception age

The number of studies and methodologies used are shown in Fig. 2 (Panel A and B, “preterms”).

## Structural asymmetries in premature infants before term PCA

We found three studies investigating structural asymmetries in premature infants. A larger right hemisphere has been reported, as well as the fact that gyral complexity emerges earlier on the right, particularly in the superior temporal sulcus (Dubois et al. 2008, 2010). The same author also reported larger regions posterior and anterior to the Sylvian fissure (respectively close to planum temporale and Broca’s region) on the left side (Dubois et al. 2010). The right hippocampus showed a rightward asymmetry (Guo et al. 2015).

Results on structural brain asymmetries are shown in Fig. 3 (above, “preterms”).

## Functional asymmetries in premature infants before term PCA

We found three studies investigating endogenous cerebral activity without sensory stimulation and seven under sensory stimulation (auditory [speech and non-speech] and tactile) in premature infants. Some studies have been conducted to investigate the presence of functional asymmetries in the resting state and under stimulation.

Studies investigating cerebral activity at rest converged to identify a rightward dominance, supported by the use of various techniques (spectral and connectivity analysis of the EEG, NIRS, and DCS) (Eiselt et al. 1997; Lin et al. 2013; Cainelli et al. 2020b). Results were mainly found in central-posterior and temporal areas, but not in frontal areas, according to the slower development rate of the frontal areas (Lin et al. 2013).

In addition, the response to stimulation appeared to evoke a greater response in the right hemisphere for non-speech stimuli (such as oddball paradigms and auditory clicks) (Mento et al. 2010; Kaminska et al. 2018) or non-linguistic aspects of speech stimuli, such as prosody, but only preterm at later PCAs (Arimitsu et al. 2018). Phonemic contrast (Arimitsu et al. 2018) and consonant–vowel syllable (Maitre et al. 2014) stimulation evoked leftward dominance. Furthermore, the asymmetry correlated with communicative abilities at 6 and 12 months (Maitre et al. 2014). However, a rightward asymmetry has also been found to detect repetitive syllabic stimuli (Daneshvarfard et al. 2019). Orocutaneous stimulation gave divergent results in terms of left (Barlow et al. 2014) or right (Song et al. 2014) dominance.

Results on functional brain asymmetries are shown in Fig. 3 (below, “preterms”).

## The case of premature infants at term-corrected age

Preterm and full-term infants demonstrated rightward hippocampal asymmetry (Thompson et al. 2009; Guo et al. 2015), but preterm infants tended to have less asymmetrical hippocampi than full-term infants (Thompson et al. 2009). A white-matter tract investigation using DTI in premature infants at term showed a leftward asymmetry in the parieto-temporal part of the superior longitudinal fasciculus and a trend toward leftward asymmetry in diffusion indices in the corticospinal tract. Furthermore, a leftward volume asymmetry has been found in the motor part of the superior thalamic radiations (Li et al. 2015).

From a functional point of view, it has been reported that premature infants at term-corrected age show lower spectra compared to full-term neonates in specific regions, particularly in the left parasagittal and the sagittal regions (Scher et al. 1997). Furthermore, they showed lower coherence in the left frontopolar–centrotemporal and right occipital–centrotemporal regions in the beta band during active sleep (González et al. 2011). Using fMRI connectivity measures at rest in very preterm subjects (birth weight 500–1500 g) at term equivalent age, premature neonates revealed more significant differences in cerebral lateralization in the left hemisphere language regions than controls (Kwon et al. 2015).

Results on brain asymmetries are shown in Fig. 3 (above, “preterms”).

## Sex differences

Of the 57 works, 22 explored the presence of sexual dimorphism in hemispheric asymmetries explicitly (10 on full-term infants, 6 on foetuses, and 6 on premature infants). Only five found sex-based differences. The differences detected are difficult to compare because studies focused on different targets, but the results appear to be quite contrasting. For example, it has found a more predominant leftward lateralization during gestation for males (Kivilevitch et al. 2010), rightward in premature infants for females (Dubois et al. 2008), and males (Lin et al. 2012).

## Discussion

We performed a scoping review of the existing literature on hemispheric asymmetries in the first brain development phases. We reviewed studies using neuroimaging methods, which provide direct evidence on hemispheric, structural, and/or functional asymmetries, in full-term neonates, foetuses during pregnancy, and premature infants, both at term PCA and before. Given the low number of studies, we did not select a specific year range. Rather, we collected all the available evidence, yielding 57 studies.

The reviewed literature shows high variability in techniques and methodological procedures. Most studies based on the neonatal period and gestation were structural investigations, while most of those conducted in premature infants were functional. Finally, we searched for sexual dimorphisms, but the large majority of the studies did not find differences in hemispheric asymmetries in males and females.

A high discordance between results emerged in reviewing studies on structural asymmetries. The discordance is not explainable by a low number of participants because most studies had large sample sizes. Furthermore, the participant numbers between the studies may differ significantly. Brain asymmetries may be quite small, depending on the measurement (for example, see Kong et al. 2018), which in turn might lead to differences between studies, especially when the sample sizes differ. The temporal lobe is the most studied cerebral structure—the first neuropathological reports describe a larger left temporal hemisphere. Despite incomplete agreement between studies, evidence supports a larger planum temporale on the left side and a deeper superior temporal sulcus on the right. It has been reported that during primary gyrogenesis, the right superior temporal sulcus undergoes cortical folding earlier (Rajagopalan et al. 2011, 2012; Kasprian et al. 2011; Habas et al. 2012) and shows larger gyral complexity (Dubois et al. 2008, 2010). In addition, here, differences in sample size may perhaps justify some differences between studies.

The temporal planum on the left is often included in the Wernicke’s area (Tremblay and Dick 2016), responsible for understanding spoken language. These data are in line with studies on the adult brain, showing that the temporal planum is more pronounced on the left than on the right in most individuals (Geschwind and Levitsky 1968).

Most results on the parieto-occipital cortex and subcortical grey matter exhibit a rightward asymmetry in full-term and premature infants. Brain structures with slower developmental rates, such as white matter and frontal lobes, have been poorly investigated, and the results are even more discordant.

Studies during gestation are scarce (*n* = 11), and all but one structural. Compared to studies on premature infants, those on foetuses are mainly conducted in earlier gestational weeks (late second and initial third trimester of gestation vs. the end of the third), when the brain is very immature. Therefore, anatomical investigations are less refined. As asymmetry emergence is mainly characterized by enlarging of the regions surrounding the Sylvian fissure in the left hemisphere (Dubois et al. 2008, 2010; Liu et al. 2010; Habas et al. 2012), these studies usually detected only a global enlargement of the left hemisphere. Results agree substantially with the first reports on the left-sided temporal lobe being significantly larger in post-mortem foetuses (Witelson and Pallie 1973; Chi et al. 1977), a morphological asymmetry already present from the 29th week of gestation (Wada et al. 1975).

Unlike data on structural asymmetries, functional data obtained in full-term infants, premature infants, and foetuses show a more harmonious pattern of results. Studies converge to identify a leftward dominance for speech stimuli; interestingly, this functional asymmetry correlates with communicative abilities at 6 and 12 months (Maitre et al. 2014), supporting the specificity of the left temporal lobe for language.

The other main finding of our revision is an overall dominance of the right hemisphere in all other functional conditions: sensory stimulations, non-linguistic characteristics of speech, and endogenous activity obtained during a resting state. The dominance of the right hemisphere for all conditions except linguistic stimuli is in line with the right-hemisphere conservatism theory (Geschwind and Galaburda 1985), stating that the right hemisphere develops earlier and that its development is, therefore, less subject to external influences. The delay in maturation of the left hemisphere may allow higher plasticity in terms of environmental stimulation, such as language exposure and motor movements (Dubois et al. 2008). Compelling support has also been provided by Sun et al. (2005), who found significant asymmetries of gene expression in embryos as early as 12-week gestational age.

In humans, as in animals, the right hemisphere sustains those functions necessary to survive, including visuospatial or emotional processes, which render its early development adaptive (Geschwind and Galaburda 1985). It has been shown, for example, that the right hemisphere systematically prevails over the left hemisphere in recognition of faces and facial expressions, mental rotation, and para-verbal stimuli, such as prosody and recognition of the connotative and affective tone of spoken language (see, e.g., George et al. 1996). Furthermore, right lateralization is established for the ventral frontoparietal network, which acts as a detector of relevant stimuli (especially if salient and unexpected) in a model by Corbetta and Shulman (2002). All these functions are crucial for a neonate.

Initial investigations of hemispheric asymmetry consisted of post-mortem explorations of aborted foetuses or dead newborns. Advances have come more quickly in the era of neuroimaging, overcoming the problem of small sample sizes and increasing the data availability also of healthy subjects; however, the increase in available data has rather complicated the evidence. Our data suggest that functional asymmetry regarding language is correlated with the perisylvian regions’ structural asymmetry, but other associations between structural and functional findings are hard to establish. It is noteworthy that the relationship between structural and functional asymmetries is still far from being fully characterized also in adult studies (Dos Santos Sequeira et al. 2006). Rather more in agreeance are results on functionally critical morphological asymmetries, such as microstructural organization. For example, dendritic arborisation is usually greater in the language areas of left hemisphere than in the corresponding areas on the right (Scheibel et al. 1985).

Our review also highlights another dissociation between structural and functional results: while a broad agreement was found in functional studies, structural findings showed a low concordance among themselves. This appears particularly curious, considering the higher variability in techniques (EEG, fMRI, ERP, EP, NIRS, and MEG) and conditions (at rest or under stimulation) used in functional studies compared to structural ones. The reason is unclear, but functional studies may allow the cerebral functionality to emerge using appropriate tasks targeted to the immature brain. On the other side, structural investigations are static photographs of the whole brain. They provide information on the areas that mature later and exhibit higher plasticity in terms of experience, which may justify a higher inter-individual variability.

Interestingly, studies on older children and adults born prematurely have shown that volumetric and microstructural abnormalities are scarcely associated with neurodevelopment outcomes (Nosarti et al. 2008; Mathur et al. 2010; Seghier and Hüppi 2010). In contrast, functional connectivity data have been highly correlated with intelligence and task performance measures (Seeley et al. 2007; Van Den Heuvel et al. 2009; Nosarti et al. 2009; Myers et al. 2010). Connectivity analysis has been suggested to be particularly revealing when assessing hemispheric specialization (Stephan et al. 2007).

Finally, our review of the literature showed that premature infants had altered asymmetry measures compared to full-term infants, also in the absence of other risk factors. These data are in line with other studies on the developmental trajectories of premature infants (Suppiej et al. 2015, 2017; Cainelli et al. 2020a, 2021). Furthermore, lateralization abnormalities have been shown to persist over the long term: prematurely born adolescents exhibit fundamental alterations in the cerebral lateralization for language that significantly correlate with language scores (Wilke et al. 2014; Scheinost et al. 2015). Lateralization is implicated in language development, handedness, and higher-order reasoning and processing (Steinmetz et al. 1991; Turner et al. 2015). Therefore, the study of early abnormalities may help explain typical neurodevelopment and the origin of disorders, given the increased vulnerability to many extrinsic and intrinsic influences at this developmental phase (Andersen 2003). Abnormalities in structural and functional lateralization are suspected of contributing to various neuropathologies in humans, as several neurodevelopmental pathologies, such as schizophrenia (Oertel-Knöchel and Linden 2011; Ribolsi et al. 2014), obsessive–compulsive disorder (Rao et al. 2015), autism spectrum disorder (Gabard-Durnam et al. 2015), attention-deficit and hyperactivity disorder (Sigi Hale et al. 2014), and dyslexia (Brandler and Paracchini 2014) are associated with atypical patterns of functional and structural asymmetries.

Scoping reviews are useful for examining emerging evidence when it is still unclear what other, more specific questions can be posed and valuably addressed by a more precise systematic review (for guidance, see Tricco et al. 2018; Munn et al. 2018). As such, scoping reviews cannot uncover the international evidence, confirm current practice/address or any variation/identify new practices, identify and investigate conflicting results, produce statements to guide decision-making, as systematic reviews do (Munn et al. 2018). In particular, the provision of implications for practice is a key feature of systematic reviews that lacks in scoping reviews, given its absence of an assessment of methodological limitations or risk of bias of the evidence.

Investigating the emergence of early asymmetries, scoping review is the best choice, given the current literature in this research field. We hope that our work may be the starting point for future research and systematic reviews, which may, respectively, address unexplored areas or systematically reviews specific questions. For example, our work highlights several fields that request further investigation: sexual dysmorphisms, the development of structural and functional brain asymmetries in healthy foetuses, the presence of asymmetries in less explored brain regions, such as frontal and subcortical structures.

In conclusion, all but one study agreed on the existence of hemispheric asymmetry as early as the first appearance of cerebral structures. Functional asymmetry for language is correlated with the structural asymmetry of perisylvian regions. However, studies do not agree on the developmental direction, and the structural locations of several other asymmetries emerged, while most consistent results came from functional data. Globally, data related to a general dominance of the right hemisphere, accompanied by a selective leftward dominance for language, are in line with the assumption of an early-maturing right hemisphere and less genetic control over the left hemisphere, which would be influenced more by the in utero environment (Geschwind and Galaburda 1985; Geschwind et al. 2002). Understanding normative development is necessary to understand abnormalities in diseases and how they affect early-life experiences. We are still far away from a clear understanding of developmental trajectories and the significance of potential disorders later in neurodevelopment.

## Supplementary Information

Below is the link to the electronic supplementary material.

Supplementary file1 (BIB 1 kb)

## Data Availability

Not applicable.
